# Supramolecular attack particle: the way cytotoxic T lymphocytes kill target cells

**DOI:** 10.1038/s41392-020-00319-z

**Published:** 2020-09-21

**Authors:** Jiaqi Liu, Yanqi Ye, Lulu Cai

**Affiliations:** 1 Personalized Drug Therapy Key Laboratory of Sichuan Province, Department of Pharmacy, Sichuan Provincial People’s Hospital, University of Electronic Science and Technology of China, Chengdu, 610072 China; 2 Zenomics Inc., California NanoSystems Institute, University of California, Los Angeles, CA 90095 USA

**Keywords:** Molecular medicine, Breast cancer

**Fig. 1:**
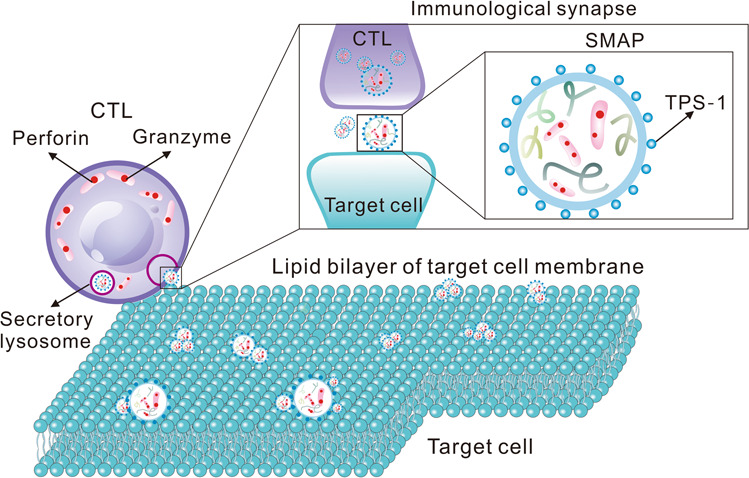
The cytotoxic substances in CTL, perforin, and granzyme, assembled into supramolecular attack particles (SMAPs), which were released toward and remained attached to the target cell membrane after the CTL removal. SMAPs, ~120 nm in diameter, have a dense shell that includes TSP-1 and a core of perforin, granzymes, and other cytotoxic proteins

A very recent study by Bálint et al. published in *Science* deciphered in detail how cytotoxic T lymphocytes (CTLs) kill target cells.^1^ They found that perforin and pellet enzyme—the cytotoxic substances in CTLs—combined and assembled into supramolecular attack particles (SMAPs). The SMAPs were subsequently released toward the cell membrane of the target cell. The shell of SMAP was rich in glycoproteins that enabled its stability to maintain the cell-killing activity for hours, in which the thrombospondin-1 (TSP-1) was critical (Fig. 1).

There are many types of T cells, among which the CTLs play a major role in destroying virus-infected cells and cancerous cells.^2^ The killing mechanism mediated by CTLs has been studied extensively before and there are two main mechanisms. One is degranulation induced upon the target cell recognition. The CTL identifies the target cell, forms an immunological synapse (IS) and releases perforin (Prf1) and soluble granzymes (Gzmb) from secretory lysosomes (SLs). Prf1 creates holes in the membrane of target cells through which Gzmb enters to initiate various apoptosis pathways.^3,4^ Tamzalit et al. found that even in the absence of perforin, the CTL could initiate necrosis and kill target cells by its mechanical movement.^5^ The other mechanism is by activating the self-apoptosis process of the cells. CTLs can recognize the death receptor Fas (FasL) expressed on target cells to induce programmed cell death. Despite the ongoing studies, it remains unclear on the details of killing mechanism and its relation to the secretion of cytotoxic molecules Prf1 and Gzmb.

To track how perforin and granzyme work when CTLs kill the targets, the authors labeled granular enzymes in CTLs and glycoproteins in SLs with different fluorescein in this work. After in contact with the CTLs, the target cells showed intense double-positive puncta on the surface. These labeled multiprotein structures were defined as SMAPs. With a supported lipid bilayers (SLB) to simulate the target cell, the authors investigated the kinetics of SMAPs release. The SLs in the CTLs rapidly accumulated at the IS after the CTLs were incubated on the SLB. It was rapidly followed by the appearance of Gzmb puncta in the SLB and the attachment of SMAPs to the surface of the simulated target cell.

After CTL removal, Prf1^+^ and Gzmb^+^ SMAPs were found to remain attached on the SLB. The authors then tested the stability of SMAPs after being released. After 20 minutes of CTL removal, the SMAPs still existed in the IS and maintained the activity of killing the target cell. The authors further investigated the SMAPs captured on SLB by mass spectrometry. More than 285 proteins were present in SMAPs, including peptides from Prf1 and Gzmb. This result was also confirmed by SDS-PAGE and immunoblotting. Among these proteins, TSP-1 caught the authors’ attention due to its signature Ca^2+^ binding repeats, which corresponded to the well-established Ca^2+^ dependent steps in CTL mediated killing. After knocking out TSP-1 by 60%, the CTLs killing efficacy reduced by 30%. These results indicated that the TSP-1 component in SMAP played an important role in the process of CTL killing target cells.

Furthermore, they investigated SMAPs from a microscopic perspective. Each IS contained about 27 SMAPs with a diameter of about 120 nm. TSP-1 was found to be co-localized with Prf1 and Gzmb, either in the secreted SMAPs or in the CTLs. It was likely that molecules such as Prf1 and Gzmb were assembled into SMAPs and waited to be released in the secretory lysosome of CTLs. The distribution of TSP-1 in SMAP was also consistent with the glycoprotein shell, which turned out to be a component of the SMAP glycoprotein shell. All these results indicated the specific construction of SMAP—a mixed particle with a diameter of about 120 nm consisting of a dense shell with TSP-1, and a core structure containing Prf1, Gzmb, and other cytotoxic substances.

In summary, the study by Bálint and coworkers innovatively illustrated how CTLs attack the target cells by secreting SMAPs containing TSP-1 and deadly chemicals such as Prf1 and Gzmb. They revealed the details and mechanisms that CTLs kill target cells, further enhancing our understanding of CTLs. Besides, SMAPs contain many immune regulatory factors that may regulate the immunity in the tumor microenvironment. The key protein TSP-1 has a binding site for “don’t eat me” signal CD47 to myeloid cells, so that any cell escaped from SMAPs may be culled by phagocytosis. All of these findings provide implications for developing new ideas for cancer immunotherapy.
